# Perceived academic stress is associated with reactive aggression through anger rumination and lower self-control in Chinese senior high school students

**DOI:** 10.3389/fpsyg.2026.1915269

**Published:** 2026-10-21

**Authors:** Xinyuan Li, Guanming Huang, Liting Li, Jie Xiong, Zhuoying Lin, Shaojuan Hu

**Affiliations:** School of Physical Education and Sports Science, Hengyang Normal University, Hengyang, Hunan, China

**Keywords:** anger rumination, Chinese senior high school students, perceived academic stress, reactive aggression, self-control, serial mediation

## Abstract

**Background:**

Senior high school is a developmental period marked by intensive academic demands, social evaluation, and competition for further education. Perceived academic stress may be associated with adolescents’ reactive aggression, but the potential cognitive–emotional and self-regulatory pathways involved in this association remain insufficiently integrated.

**Purpose:**

This study examined whether anger rumination and self-control showed independent and serial statistical mediating effects in the association between perceived academic stress and reactive aggression.

**Methods:**

A cross-sectional questionnaire design was used. The final sample comprised 2,328 students aged 15 years or older in Grades 10–12 from three regular senior high schools in Hengyang, Hunan Province, China. Confirmatory factor analyses were conducted for the scales and for a correlated four-construct measurement model. Descriptive, correlation, group-difference, and regression analyses were performed using composite scores. Independent and serial statistical mediation models were tested using PROCESS Models 4 and 6, respectively. Sex, age, and grade were included as covariates, and indirect effects were estimated using 5,000 bootstrap resamples and 95% confidence intervals.

**Results:**

Perceived academic stress was positively associated with reactive aggression (*B* = 0.303, *β* = 0.250, *p* < 0.001). Separate mediation analyses showed significant partial statistical mediation involving anger rumination (indirect effect = 0.098, 95% bootstrap confidence interval [0.0725, 0.1232]) and self-control (indirect effect = 0.052, 95% bootstrap confidence interval [0.0375, 0.0676]). Serial mediation analysis further showed a significant serial statistical indirect effect involving higher anger rumination and lower self-control (indirect effect = 0.013, 95% bootstrap confidence interval [0.0083, 0.0184]). The direct effect of perceived academic stress on reactive aggression remained significant after the mediators were included.

**Conclusion:**

Perceived academic stress was positively associated with reactive aggression among Chinese senior high school students. Separate and serial statistical indirect effects involving anger rumination and self-control were observed. These findings suggest that the association between academic stress and reactive aggression may be linked to anger-related perseverative processing and lower self-control. Given the cross-sectional design, the findings should be interpreted as statistical associations and indirect effects consistent with the hypothesized theoretical order, rather than as evidence of temporal ordering or causal relationships.

## 1 Introduction

Senior high school is a developmental period marked by intensive academic demands, social evaluation, and competition for further education. Students must cope with coursework, examination pressure, and expectations regarding further educational advancement while adapting to school life and peer interactions during rapid physical and psychological development. In this context, perceived academic stress (PAS) has increasingly been recognized as an important school-contextual factor in adolescent psychological adjustment. Review evidence indicates that academic stress is common during senior high school and has been associated with psychological distress, anxiety, and depressive symptoms (Wuthrich et al., 2020; Steare et al., 2023). At the same time, reactive aggression (RA), defined as emotionally driven and impulsive aggressive responses that are more likely to occur when individuals are provoked, frustrated, or threatened, may be associated with poorer peer relationships and school adjustment and may complicate the prevention and management of behavioral risks among adolescents. In recent years, student mental health promotion and behavioral risk prevention have received considerable attention at the national policy level. In 2023, the Ministry of Education of the People’s Republic of China and 16 other government departments issued the Special Action Plan for Comprehensively Strengthening and Improving Student Mental Health in the New Era (2023–2025), which called for improving the student mental health support system and strengthening monitoring and early warning, counseling services, and intervention and response procedures. This policy context further underscores the practical need to examine academic stress and associated behavioral risks in Chinese school settings (Ministry of Education of the People’s Republic of China et al., 2023).

Prior research has suggested a significant positive association between academic stress and aggression (Li C. et al., 2023). However, the specific cognitive–emotional and self-regulatory pathways involved in the association between academic stress and reactive aggression among senior high school students remain insufficiently integrated. According to the perseverative cognition hypothesis, stressful events do not necessarily dissipate once external circumstances end; instead, they may be prolonged and maintained internally through perseverative cognitive processing. From this perspective, higher PAS may be associated with greater anger rumination (AR) (Brosschot et al., 2006). Similarly, the strength model of self-control posits that self-control relies on limited resources and that sustained demands for control, inhibition, and emotion regulation may deplete these resources. Thus, higher PAS may also be associated with lower self-control (SC) (Baumeister et al., 2007). Accordingly, this study aimed to (1) examine the association between PAS and RA; (2) test whether AR and SC showed independent statistical mediating effects in this association; and (3) examine whether AR and SC formed a serial statistical mediation pathway. These analyses were intended to provide empirical evidence for understanding the pattern of associations among school stress, cognitive–emotional processing, self-regulatory resources, and behavioral risk in adolescents. Because the study used cross-sectional questionnaire data, the hypothesized paths were specified only to characterize statistical associations consistent with the proposed theoretical order and were not intended to establish temporal ordering or causal mechanisms.

## 2 Literature review and research hypotheses

### 2.1 Perceived academic stress and reactive aggression

Perceived academic stress refers to adolescents’ subjective experience of stress in school learning contexts, including coursework load, performance pressure, competition, and self-expectations (Sun et al., 2011), whereas reactive aggression refers mainly to anger-driven and impulsive aggressive responses that individuals display when they are provoked, frustrated, or threatened (Raine et al., 2006). During senior high school, academic stress is not merely a burden arising from learning tasks. Rather, it may represent a highly evaluative stress experience shaped by multiple personal, familial, and school-related factors, such as school emphasis on academic achievement, parental expectations, personal achievement concerns, and an orientation toward further education (Chyu and Chen, 2022). Relevant reviews have also indicated that academic stress is common during senior high school and is consistently associated with a range of adverse psychological outcomes (Wuthrich et al., 2020; Steare et al., 2023). With respect to behavioral outcomes, previous research has found a significant positive association between academic stress and aggression (Li C. et al., 2023). School stress has also been associated with externalizing problem behaviors such as bullying (Luo et al., 2023), and, in the Chinese cultural context, perceived parental academic pressure has been linked to poorer psychological adaptation among adolescents (Xiong et al., 2024). Theoretically, the frustration–aggression hypothesis posits that when individuals experience external demands as goal obstruction, evaluative threat, and persistent frustration, they are more likely to experience negative arousal and display aggressive responses (Berkowitz, 1989). General strain theory further emphasizes that negative emotions such as anger activated by strain can increase the likelihood of deviant and aggressive behaviors (Agnew, 1992). Accordingly, among Chinese senior high school students, higher perceived academic stress may be associated with higher reactive aggression. Therefore, this study proposed the following hypothesis:

*H1*: Perceived academic stress is significantly and positively associated with reactive aggression.

### 2.2 Mediating role of anger rumination

Anger rumination refers to the tendency to repeatedly think about and persistently process anger-related events, feelings of anger, and their causes and consequences. It differs not only from general rumination but also from sadness rumination, and it shows stronger content specificity in relation to aggressive behavior (Peled and Moretti, 2010; Harmon et al., 2019). Previous research has further indicated that anger rumination shows relatively specific associations with different forms of aggression (Anestis et al., 2009). On this basis, anger rumination may be an important cognitive–emotional variable involved in the association between perceived academic stress and reactive aggression. According to the perseverative cognition hypothesis, stressful events do not necessarily dissipate once external circumstances end; rather, they may be prolonged and maintained internally through perseverative cognitive processing (Brosschot et al., 2006). Although studies directly focusing on academic stress and anger rumination remain relatively limited, existing bridging evidence indicates that parental academic pressure is significantly associated with negative psychological outcomes such as anxiety and depression (Quach et al., 2015), that academic stress has been linked to higher levels of burnout in models involving academic anxiety (Gao, 2023), and that academic pressure is also related to subsequent anxiety and stress-related physiological responses (Hua et al., 2023). Meanwhile, more general stress exposure has been linked to a higher risk of aggression in models involving emotion dysregulation (Herts et al., 2012). Together, these strands of bridging evidence suggest that academic stress, as a highly evaluative and high-arousal school stressor, may be associated with higher anger rumination in the context of sustained negative cognitive–emotional processing. On the other hand, the General Aggression Model posits that persistent hostile cognition, negative affect, and arousal increase the likelihood of aggressive behavior in provocative situations (Allen et al., 2018). Studies of adolescents have also shown that anger rumination is consistently associated with overt aggression and relational aggression (Smith et al., 2016), suggesting that anger rumination may likewise be associated with reactive aggression, which is characterized by emotional reactivity and provocation. Accordingly, among Chinese senior high school students, higher perceived academic stress may be associated with higher anger rumination, which in turn may be associated with higher reactive aggression. Therefore, this study proposed the following hypothesis:

*H2*: Higher perceived academic stress is statistically indirectly associated with higher reactive aggression through higher anger rumination.

### 2.3 Mediating role of self-control

Self-control refers to the capacity to regulate thoughts, emotions, and behaviors and to inhibit immediate impulsive responses in pursuit of long-term goals. It is regarded as an important protective self-regulatory resource that is consistently associated with broader adaptive behavioral outcomes (de Ridder et al., 2012), and it has developmental significance across time and domains (Moffitt et al., 2011). On this basis, self-control may represent an important self-regulatory variable involved in the association between perceived academic stress and reactive aggression. According to the strength model of self-control, self-control depends on limited resources, and sustained demands for control, inhibition, and regulation may occupy and deplete these resources, thereby weakening subsequent control capacity (Baumeister et al., 2007). A related meta-analysis also showed that prior exertion of control was associated with poorer subsequent self-control performance, providing evidence for the overall effect of ego depletion (Hagger et al., 2010). Although research directly focusing on academic stress and self-control remains limited, existing bridging evidence suggests that academic stress, aggression, and self-control may be interrelated in longitudinal models of adolescent behavioral risk, particularly in relation to pathological gaming (Kim et al., 2025). This evidence is consistent with the possibility that academic stress may be associated with adverse behavioral outcomes through weakened self-regulatory resources. On the other hand, the potential association between self-control and reactive aggression can be explained by the Instigation–Impellance–Inhibition Theory (I^3^ Theory): when individuals are in provocative, frustrating, or high-arousal situations, weaker inhibition may make it more difficult to suppress immediate retaliatory impulses, thereby making reactive aggression more likely (Finkel, 2014). Relevant reviews further suggest that failures of self-control are closely associated with aggressive behavior, whereas stronger control capacity helps inhibit aggressive responses (Denson et al., 2012; DeWall et al., 2011). Accordingly, among Chinese senior high school students, higher perceived academic stress may be associated with lower self-control, which in turn may be associated with higher reactive aggression. Therefore, this study proposed the following hypothesis:

*H3*: Higher perceived academic stress is statistically indirectly associated with higher reactive aggression through lower self-control.

### 2.4 Serial mediation effect of anger rumination and self-control

Existing research suggests that anger rumination and self-control may form a serial statistical mediation pathway between perceived academic stress and reactive aggression. As a persistent form of negative cognitive processing, rumination is regarded as an important form of maladaptive emotion regulation and is consistently associated with aggression (Navas-Casado et al., 2023). According to the perseverative cognition hypothesis, stress does not necessarily dissipate once external events end; rather, it may be prolonged and maintained within the individual through conscious or unconscious perseverative cognitive processing (Brosschot et al., 2010). Related research has further linked facets of rumination and trait anxiety to resting heart rate variability, a physiological indicator relevant to regulatory functioning (Williams et al., 2017). A meta-analysis also found that perseverative cognition was accompanied by higher blood pressure, heart rate, and cortisol levels, as well as lower heart rate variability (Ottaviani et al., 2016). Together, these strands of bridging evidence suggest that persistent anger rumination may not only maintain negative arousal but also further consume regulatory resources and weaken executive and control functions. This interpretation is consistent with the Self-Regulatory Executive Function Model, which provides a framework for understanding how sustained cognitive–attentional processing may be linked to self-regulatory functioning in emotional contexts (Wells and Matthews, 1996).

On this basis, previous studies have shown that stress may be associated with more severe adverse outcomes through rumination (Ye et al., 2020), that academic stress may be associated with self-harm behaviors through depression and anxiety (Chen et al., 2023), and that academic burden may be longitudinally associated with poorer emotional outcomes among adolescents through mechanisms such as loneliness, physical activity, and sleep (Wang et al., 2025). Evidence more closely aligned with the sequence examined in the present study has also shown that academic pressure can be associated with problem behavior through pathways involving lower self-control, among other factors (Jiang et al., 2022). Meanwhile, the Instigation–Impellance–Inhibition Theory further suggests that individuals are more likely to display aggressive behavior when instigating and impelling forces are stronger and inhibitory functioning is relatively weaker (Finkel and Hall, 2018). Accordingly, among Chinese senior high school students, higher perceived academic stress may first be associated with higher anger rumination, which may then be associated with lower self-control and, in the hypothesized sequence, with higher reactive aggression. The serial order of anger rumination and self-control specified in this study is theory-driven and does not imply that alternative orders are empirically impossible. Therefore, this study proposed the following hypothesis:

*H4*: Higher perceived academic stress is statistically indirectly associated with higher reactive aggression through a serial pathway involving higher anger rumination and lower self-control.

Taken together, the reviewed literature supports the theory-driven specification of the present study: perceived academic stress may be positively associated with reactive aggression, and anger rumination and self-control may show independent and serial statistical mediating effects in this association. Accordingly, this study developed a hypothesized research model (Figure 1) and a theory-supporting framework model (Figure 2). Figure 1 presents the hypothesized associations and path structure among perceived academic stress, anger rumination, self-control, and reactive aggression, whereas Figure 2 summarizes the main theoretical basis for each path and the logical connections among these paths. The present study therefore examined: (1) the association between perceived academic stress and reactive aggression; (2) the statistical mediating effect of anger rumination in this association; (3) the statistical mediating effect of self-control in this association; and (4) the serial statistical mediating effect of anger rumination and self-control in this association.

**Figure 1:**
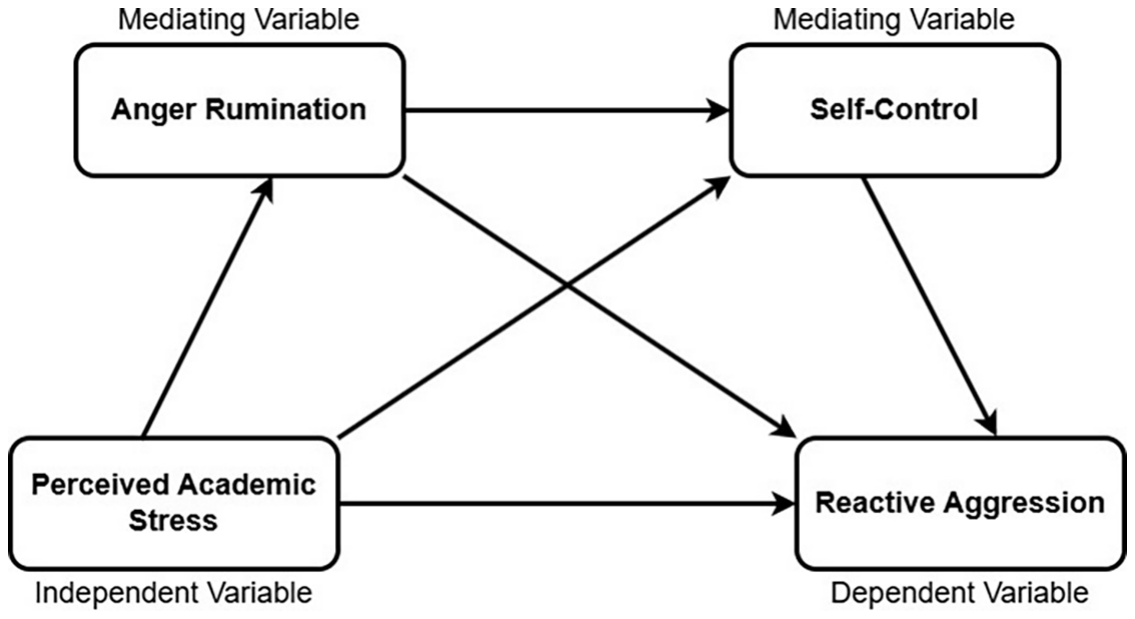
Hypothesized research model of the associations among perceived academic stress, anger rumination, self-control, and reactive aggression. Arrows represent the hypothesized statistical paths and do not establish temporal or causal ordering.

**Figure 2:**
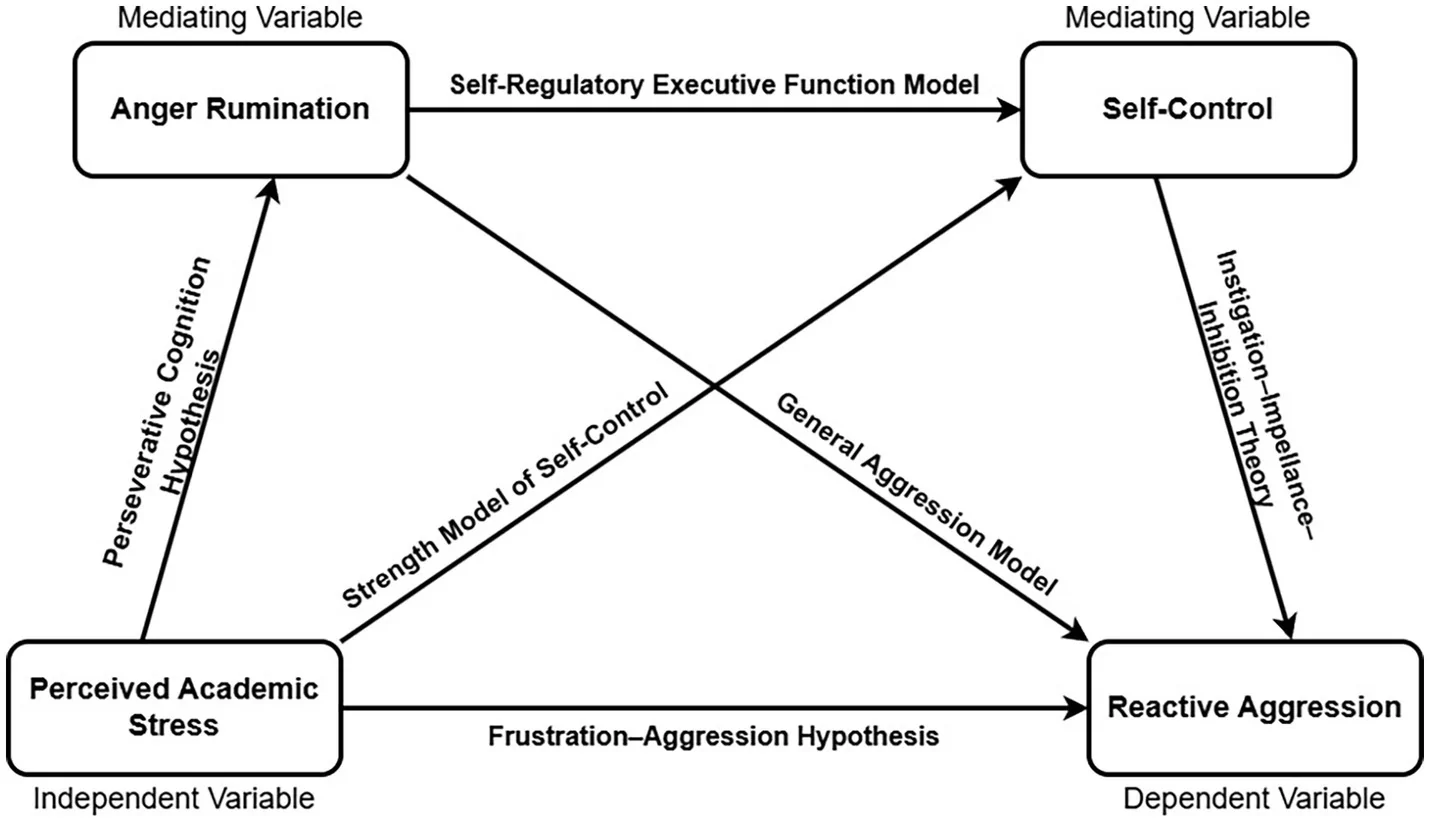
Theory-supporting framework model for the hypothesized paths among perceived academic stress, anger rumination, self-control, and reactive aggression. The labels on the arrows indicate the theoretical basis proposed for each corresponding path.

## 3 Materials and methods

### 3.1 Participants

A questionnaire survey was conducted from May 8 to May 20, 2026, at three regular senior high schools in Hengyang, Hunan Province, China. The participants were enrolled students in Grades 10, 11, and 12. Using convenience sampling, the three schools were selected based on accessibility and willingness to participate; faculty members at the authors’ institution contacted school administrators, and the principals provided oral permission. After being briefed by the research team, homeroom teachers explained the study and distributed and collected the anonymous paper questionnaires during self-study periods. Students completed the questionnaires independently and could decline or discontinue participation without adverse consequences; research staff were not present during completion, and homeroom teachers did not inspect individual responses. The inclusion criteria were enrollment in Grade 10, Grade 11, or Grade 12 at regular senior high schools in Hengyang during the survey period and completion of all required questionnaire items. The exclusion criteria were a questionnaire completion time of less than 5 min, missing responses, missing key information, or evidently patterned or careless responses. The 5-min threshold and response-quality checks were used as data quality criteria to exclude responses that were unlikely to reflect careful reading and completion of all scale items. A total of 2,500 questionnaires were distributed. Of the 172 excluded questionnaires, 43 were completed in less than 5 min, 85 contained missing responses or key information, and 44 showed evidently patterned or careless responding. The final sample comprised 2,328 valid responses, yielding a valid response rate of 93.12%. Among the participants, 1,194 were male students (51.3%) and 1,134 were female students (48.7%); 256 were aged 15 years (11.0%), 617 were aged 16 years (26.5%), 770 were aged 17 years (33.1%), and 685 were aged 18 years or older (29.4%); 768 were in Grade 10 (33.0%), 759 were in Grade 11 (32.6%), and 801 were in Grade 12 (34.4%).

The study protocol was approved by the Academic Committee of Hengyang Normal University (ethics approval no. 2026LLLW015) and was conducted in accordance with the ethical principles of the Declaration of Helsinki. For participants younger than 18 years, parents or legal guardians were informed in advance through class-based parent communication groups, and no objections to participation were received. Students younger than 18 years indicated assent, whereas participants aged 18 years or older provided informed consent, by ticking the participation statement before completing the questionnaire. Questionnaires were anonymous, and individual responses were kept confidential and used only for academic research.

### 3.2 Measures

#### 3.2.1 Perceived academic stress

The Educational Stress Scale for Adolescents (ESSA; Sun et al., 2011) was used to measure perceived academic stress among senior high school students. The scale consists of 16 items and includes five dimensions: despondency/helplessness, academic workload, study pressure, worry about grades, and self-expectation stress. An example item is “I feel there is too much homework.” In this study, items were rated on a 5-point response scale, and the mean item score was used as the scale score, with higher scores indicating higher levels of perceived academic stress. In the present sample of 2,328 participants, Cronbach’s *α* was 0.820 for despondency/helplessness, 0.784 for academic workload, 0.844 for study pressure, 0.805 for worry about grades, and 0.822 for self-expectation stress. A second-order CFA showed good model fit, *χ*^2^(99) = 181.034, *p* < 0.001, *χ*^2^/df = 1.829, RMSEA = 0.019, 90% CI [0.014, 0.023], CFI = 0.995, and TLI = 0.994, supporting the prespecified factor structure in the present sample.

#### 3.2.2 Anger rumination

A Chinese version of the Anger Rumination Scale (ARS; Sukhodolsky et al., 2001), translated with the assistance of a university faculty member with extensive experience in English-language teaching, was used to measure anger rumination among senior high school students. The scale consists of 19 items and includes four dimensions: angry memories, thoughts of revenge, understanding of causes, and angry afterthoughts. An example item is “I keep thinking about events that angered me for a long time.” Participants responded according to their actual situation regarding thoughts that may occur after becoming angry or feeling offended; no fixed calendar-based recall period was specified. Before data collection, only the original 4-point response format was changed to a 5-point agreement scale to harmonize the number of response categories across the questionnaire battery (1 = strongly disagree, 2 = disagree, 3 = neither agree nor disagree, 4 = agree, and 5 = strongly agree). The mean item score was used as the scale score, with higher scores indicating higher levels of anger rumination. Because equivalence with the original 4-point response format was not tested, scores should not be directly compared across response formats. In the present sample of 2,328 participants, Cronbach’s *α* was 0.873 for angry memories, 0.826 for thoughts of revenge, 0.838 for understanding of causes, and 0.872 for angry afterthoughts. A second-order CFA showed good model fit, *χ*^2^(148) = 427.787, *p* < 0.001, *χ*^2^/df = 2.890, RMSEA = 0.029, 90% CI [0.025, 0.032], CFI = 0.986, and TLI = 0.984, supporting the prespecified factor structure in the present sample.

#### 3.2.3 Self-control

The Brief Self-Control Scale (BSCS-13; Tangney et al., 2004; Unger et al., 2016) was used to measure self-control among senior high school students. The scale consists of 13 items and is typically used as a unidimensional measure. An example item is “I am good at resisting temptation.” In this study, items were rated on a 5-point response scale, and the mean item score was used as the scale score. Items 2, 3, 4, 5, 7, 9, 10, 12, and 13 were reverse-scored, with higher scores indicating stronger self-control. In the present sample of 2,328 participants, the scale showed good internal consistency (Cronbach’s *α* = 0.937). A single-factor CFA showed good model fit, *χ*^2^(65) = 88.059, *p* = 0.030, *χ*^2^/df = 1.355, RMSEA = 0.012, 90% CI [0.004, 0.018], CFI = 0.999, and TLI = 0.998, supporting the prespecified factor structure in the present sample.

#### 3.2.4 Reactive aggression

A Chinese version of the reactive aggression subscale of the Reactive–Proactive Aggression Questionnaire (RPQ; Raine et al., 2006; Tuvblad et al., 2016), translated with the assistance of a university faculty member with extensive experience in English-language teaching, was used to measure reactive aggression among senior high school students. The subscale consists of 11 items and assesses individuals’ tendency toward impulsive aggressive responses when annoyed, provoked, frustrated, threatened, or teased. An example item is “Reacted angrily when provoked by others.” Participants responded according to their actual situation in the circumstances described by the items; no fixed calendar-based recall period was specified. The original RPQ uses a 3-point frequency response format. Before data collection, only the response format was changed to a 5-point agreement scale to harmonize the number of response categories across the questionnaire battery (1 = strongly disagree, 2 = disagree, 3 = neither agree nor disagree, 4 = agree, and 5 = strongly agree). The mean item score was used as the scale score, with higher scores indicating greater endorsement of reactive-aggression responses. Because equivalence with the original 3-point frequency format was not tested, scores should not be directly compared across response formats or interpreted as frequency-based or screening scores. In the present sample of 2,328 participants, the subscale showed good internal consistency (Cronbach’s *α* = 0.924). A single-factor CFA showed good model fit, *χ*^2^(44) = 75.141, *p* = 0.002, *χ*^2^/df = 1.708, RMSEA = 0.017, 90% CI [0.010, 0.024], CFI = 0.998, and TLI = 0.997, supporting the prespecified factor structure in the present sample.

### 3.3 Statistical analysis

Statistical analyses were performed using SPSS, AMOS, and the PROCESS macro. Frequencies, percentages, means, standard deviations, and Cronbach’s *α* were first calculated, and Harman’s single-factor test was used as a preliminary and limited diagnostic of potential common-method variance. Confirmatory factor analyses were then conducted in AMOS for each scale and for a correlated four-construct measurement model. Maximum likelihood estimation was used; PAS and AR were specified as second-order models, whereas SC and RA were specified as single-factor models, and model fit was evaluated using *χ*^2^, *χ*^2^/df, CFI, TLI, and RMSEA with 90% confidence intervals. After the prespecified factor structures were supported, a two-step analytic strategy was adopted to test theory-driven mediation models based on composite scores. First, Pearson correlation analyses, independent-samples t-tests, one-way analyses of variance, and regression analyses were conducted in SPSS using composite scores for the variables. Multicollinearity was assessed using tolerance and variance inflation factor (VIF) values. Second, mediation analyses were conducted using PROCESS: Model 4 was used separately to test the independent statistical mediating effects of anger rumination and self-control, and Model 6 was used to test their serial statistical mediating effect. Because preliminary group-difference analyses showed significant differences in the core variables by sex, age, and grade, these variables were included as covariates in the regression and mediation models. Unstandardized indirect effects were estimated from 5,000 bootstrap samples, with percentile bootstrap 95% confidence intervals. All statistical tests were two-tailed, and the significance level was set at *p* < 0.05.

## 4 Results

### 4.1 Descriptive statistics for perceived academic stress, anger rumination, self-control, and reactive aggression

Table 1 presents descriptive statistics and group-difference tests for perceived academic stress (PAS), anger rumination (AR), self-control (SC), and reactive aggression (RA). The mean scores for PAS, AR, SC, and RA were 3.29 (SD = 0.64), 3.37 (SD = 0.63), 3.21 (SD = 0.77), and 3.35 (SD = 0.77), respectively. Sex differences were statistically significant for all four variables: female students scored higher on PAS, AR, and SC, whereas male students scored higher on RA. Age and grade differences were also significant across all four variables. Overall, PAS, AR, and RA tended to be higher in older age groups and higher grade levels, whereas SC tended to be lower.

**Table 1:** Descriptive statistics (*M* ± SD) and group difference tests for PAS, AR, SC, and RA.

Group	*N*	Perceived academic stress	Anger rumination	Self-control	Reactive aggression
Male	1,194	3.25 ± 0.65	3.34 ± 0.62	3.16 ± 0.77	3.38 ± 0.78
Female	1,134	3.34 ± 0.62	3.41 ± 0.63	3.25 ± 0.77	3.32 ± 0.76
Overall	2,328	3.29 ± 0.64	3.37 ± 0.63	3.21 ± 0.77	3.35 ± 0.77
Gender differences (*t*)	–	3.169**	2.741**	2.714**	2.125*
15 years	256	3.21 ± 0.62	3.30 ± 0.61	3.36 ± 0.75	3.30 ± 0.77
16 years	617	3.25 ± 0.63	3.31 ± 0.65	3.24 ± 0.80	3.31 ± 0.77
17 years	770	3.33 ± 0.65	3.41 ± 0.62	3.18 ± 0.77	3.33 ± 0.79
18 years or older	685	3.33 ± 0.63	3.42 ± 0.61	3.16 ± 0.76	3.43 ± 0.75
Age differences (*F*)	–	3.727*	5.413**	4.813**	3.293*
Grade 10	768	3.24 ± 0.64	3.32 ± 0.61	3.27 ± 0.77	3.30 ± 0.77
Grade 11	759	3.29 ± 0.64	3.36 ± 0.62	3.20 ± 0.78	3.35 ± 0.75
Grade 12	801	3.35 ± 0.63	3.44 ± 0.64	3.15 ± 0.76	3.40 ± 0.79
Grade differences (*F*)	–	5.660**	6.649**	4.661**	3.368*

Values are presented as *M* ± SD. Gender differences were tested using independent-samples t tests; age and grade differences were tested using one-way analyses of variance (ANOVAs). Absolute *t* values are reported. *M* and SD are rounded to two decimals; *t* and *F* are rounded to three decimals. *p < 0.05, **p < 0.01, ***p < 0.001.

### 4.2 Common method bias assessment

Because all core variables were assessed using self-report questionnaires at the same time point, Harman’s single-factor test was conducted as a preliminary and limited diagnostic of potential common-method variance. A principal component analysis was conducted for all scale items, and the variance explained by the first unrotated component was examined (Podsakoff et al., 2003). The results showed that 11 factors with eigenvalues greater than 1.0 were extracted. The first factor accounted for 21.26% of the total variance, which was below the 40% rule-of-thumb threshold used in some empirical studies (Zhu and Tu, 2025). Thus, no single factor accounted for a dominant proportion of the variance; however, this limited diagnostic cannot rule out common-method variance.

### 4.3 Correlations among perceived academic stress, anger rumination, self-control, and reactive aggression

Table 2 presents the Pearson correlation results among perceived academic stress (PAS), anger rumination (AR), self-control (SC), and reactive aggression (RA). PAS was significantly and positively correlated with AR (*r* = 0.454) and RA (*r* = 0.252), and significantly and negatively correlated with SC (*r* = −0.257). AR was significantly and positively correlated with RA (*r* = 0.258) and significantly and negatively correlated with SC (*r* = −0.250). SC was also significantly and negatively correlated with RA (*r* = −0.228). All correlations were statistically significant, with all *p* values < 0.001. The directions of the correlations were generally consistent with the study hypotheses.

**Table 2:** Means, standard deviations, and correlations among study variables.

Variable	*M*	SD	1	2	3	4
Perceived academic stress (PAS)	3.29	0.64	1			
Anger rumination (AR)	3.37	0.63	0.454***	1		
Self-control (SC)	3.21	0.77	−0.257***	−0.250***	1	
Reactive aggression (RA)	3.35	0.77	0.252***	0.258***	−0.228***	1

*N* = 2,328. Pearson correlations (two-tailed) are reported. *M* and SD are rounded to two decimals; correlation coefficients are rounded to three decimals. ***p < 0.001.

### 4.4 Testing the mediating effects of anger rumination and self-control in the association between perceived academic stress and reactive aggression

A correlated four-construct measurement model was estimated in AMOS, with perceived academic stress, anger rumination, self-control, and reactive aggression specified as distinct but freely correlated latent constructs. The model showed good fit, *χ*^2^(1,637) = 2,344.931, *p* < 0.001, *χ*^2^/df = 1.432, RMSEA = 0.014, 90% CI [0.012, 0.015], CFI = 0.989, and TLI = 0.989. For the subsequent mediation analyses, perceived academic stress (PAS) was specified as the independent variable, anger rumination (AR) and self-control (SC) as mediators, and reactive aggression (RA) as the dependent variable. After controlling for sex, age, and grade, two separate PROCESS Model 4 analyses and one PROCESS Model 6 analysis were conducted to test the independent and serial statistical mediation effects. The PROCESS Model 4 results showed significant positive paths from PAS to AR (*β* = 0.446, *p* < 0.001) and from AR to RA (*β* = 0.180, *p* < 0.001). The statistical indirect effect involving AR was significant (*B* = 0.098, 95% bootstrap CI [0.0725, 0.1232]). After AR was included in the model, the direct effect of PAS on RA remained significant (*B* = 0.206, 95% CI [0.1531, 0.2587]), indicating a partial statistical mediating pattern. Similarly, the path from PAS to SC was significant and negative (*β* = −0.255, *p* < 0.001), and the path from SC to RA was significant and negative (*β* = −0.168, *p* < 0.001). The statistical indirect effect involving SC was also significant (*B* = 0.052, 95% bootstrap CI [0.0375, 0.0676]). After SC was included in the model, the direct effect of PAS on RA remained significant (*B* = 0.252, 95% CI [0.2027, 0.3003]), indicating another partial statistical mediating pattern.

Collinearity diagnostics for the SC and RA equations showed tolerance values ranging from 0.768 to 0.995 and VIF values ranging from 1.006 to 1.303, indicating no substantial multicollinearity. PROCESS Model 6 results showed that the standardized path coefficients reported in Table 3 and Figure 3 were significant for PAS → AR (*β* = 0.446, *p* < 0.001), PAS → SC (*β* = −0.181, *p* < 0.001), AR → SC (*β* = −0.165, *p* < 0.001), AR → RA (*β* = 0.156, *p* < 0.001), and SC → RA (*β* = −0.146, *p* < 0.001). After both mediators were included simultaneously, the direct effect of PAS on RA remained significant (*β* = 0.144, *p* < 0.001), and the standardized total effect was *β* = 0.250 (*p* < 0.001). Table 4 shows that all three specific indirect effects were significant: PAS → AR → RA (*B* = 0.084, 95% CI [0.0603, 0.1092]), PAS → SC → RA (*B* = 0.032, 95% CI [0.0215, 0.0445]), and PAS → AR → SC → RA (*B* = 0.013, 95% CI [0.0083, 0.0184]). In the unstandardized effect decomposition, the total indirect effect was *B* = 0.130, 95% CI [0.1033, 0.1573], accounting for 42.68% of the total unstandardized effect. The serial pathway accounted for 4.32% of the total unstandardized effect. Thus, H2, H3, and H4 were supported. Given the cross-sectional design, these mediation and serial mediation results should be interpreted as theory-consistent statistical indirect effects rather than evidence of causal mechanisms or temporal ordering.

**Table 3:** Regression analysis of the serial mediation model.

Variable	Anger rumination (AR)		Self-control (SC)		Reactive aggression (RA)		Overall effect	
	*β*	*t*	*β*	*t*	*β*	*t*	*β*	*t*
Perceived academic stress (PAS)	0.446	24.068***	−0.181	8.163***	0.144	6.436***	0.250	12.427***
Anger rumination (AR)	–	–	−0.165	7.442***	0.156	7.012***	–	–
Self-control (SC)	–	–	–	–	−0.146	7.102***	–	–
Sex	0.028	1.500	0.077	3.905***	−0.054	2.747**	−0.060	3.004**
Age	0.049	2.643**	−0.049	2.468*	0.025	1.274	0.041	2.041*
Grade	0.044	2.356*	−0.038	1.943	0.024	1.207	0.037	1.851
*R* ^2^	0.211		0.098		0.115		0.070	
*F*	155.669***		50.473***		50.161***		43.682***	

Standardized coefficients (*β*) are reported. *t* values are reported as absolute values. Sex, age, and grade were included as covariates in all models. All statistics are rounded to three decimals. *p < 0.05, **p < 0.01, ***p < 0.001.

**Figure 3:**
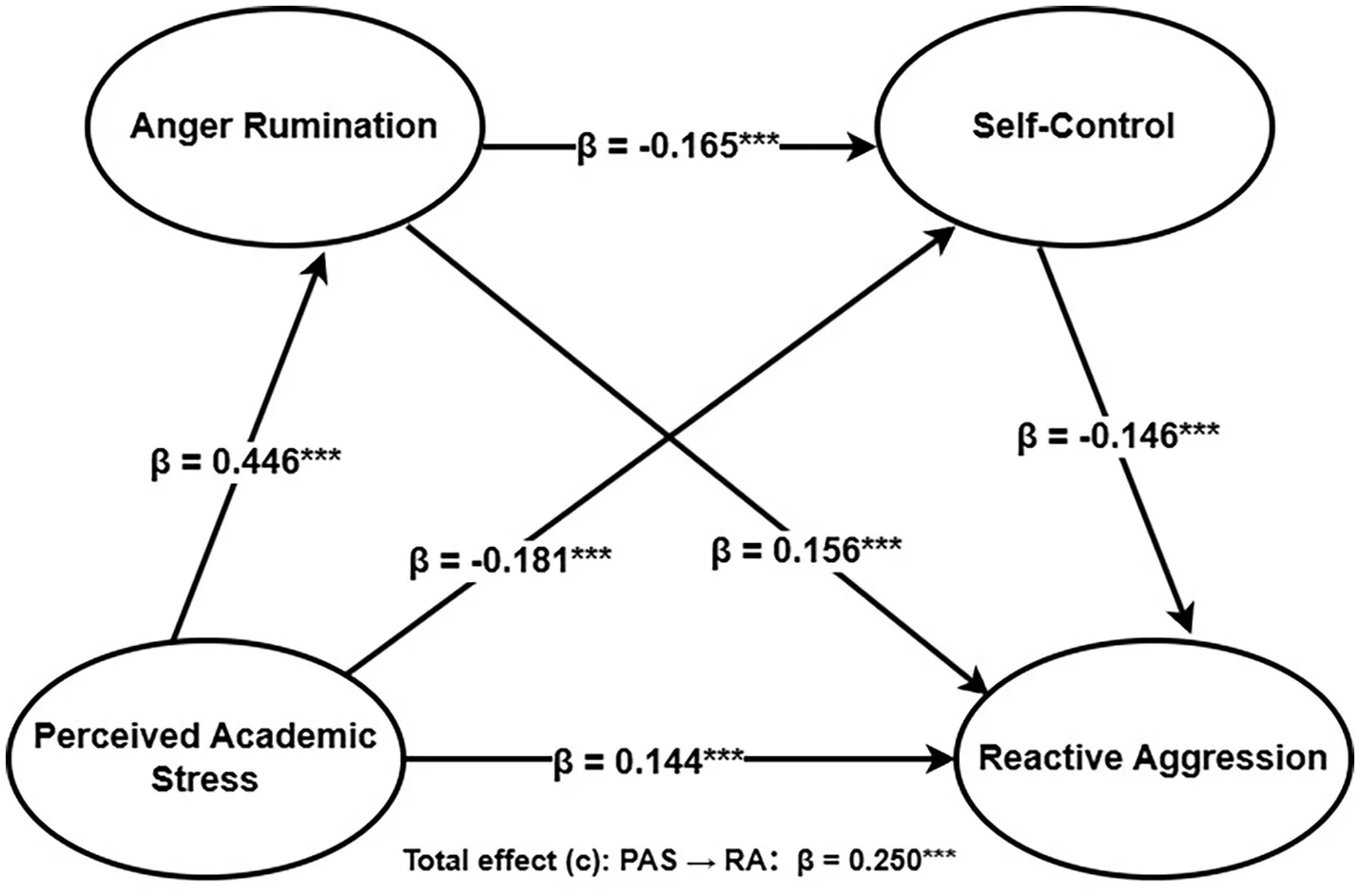
Standardized path coefficients for the serial statistical mediation model tested using PROCESS Model 6 (*N* = 2,328). Values are standardized regression coefficients (β). Sex, age, and grade were included as covariates but are not shown. The coefficient for the perceived academic stress–reactive aggression path represents the direct effect after anger rumination and self-control were included, whereas the total effect is shown below the model. ***p < 0.001.

**Table 4:** Statistical indirect effects and effect-size decomposition.

Path	Unstandardized effect (*B*)	Proportion of total effect	95% CI	
			LL	UL
PAS → AR → RA	0.084	27.82%	0.0603	0.1092
PAS → SC → RA	0.032	10.55%	0.0215	0.0445
PAS → AR → SC → RA	0.013	4.32%	0.0083	0.0184
Total indirect	0.130	42.68%	0.1033	0.1573
Direct effect (*c*′)	0.174	–	0.1209	0.2269
Total effect (*c*)	0.303	–	0.2555	0.3513

Effects are unstandardized (*B*). Indirect effects were estimated using percentile bootstrap 95% confidence intervals based on 5,000 resamples. Sex, Age, and Grade were included as covariates in all models (not shown). Proportions of the total effect were computed using unrounded estimates as (indirect effect/total effect) × 100%. CI limits are rounded to four decimals; proportions to two decimals; all other statistics to three decimals. Indirect effects are considered significant when the bootstrap 95% CI does not include zero. Bootstrap CIs are reported for indirect effects; normal-theory CIs (LLCI/ULCI) are reported for direct and total effects.

## 5 Discussion

This study examined whether perceived academic stress (PAS) is associated with reactive aggression (RA) among Chinese senior high school students and whether anger rumination (AR) and self-control (SC) characterize this association as independent and serial statistical mediators. Overall, the findings indicate that the PAS–RA association was only partially accounted for by the proposed indirect pathways. In the unstandardized effect decomposition, the total indirect effect accounted for 42.68% of the total effect, whereas the remaining 57.32% represented the direct statistical association. The full model explained 11.5% of the variance in RA (*R*^2^ = 0.115), indicating modest explanatory power. Among the indirect pathways, AR accounted for the largest proportion of the total effect, followed by SC, whereas the serial AR–SC pathway accounted for 4.32% of the total effect, indicating a small magnitude despite statistical significance. These findings suggest that the association between academic stress and reactive aggression among senior high school students cannot be fully captured by a single linear pathway. Rather, it appears to involve both a direct statistical link and indirect pathways related to anger-related perseverative processing and lower self-control. Given the cross-sectional design, these findings should be interpreted as statistical associations and indirect effects consistent with the hypothesized order, rather than as evidence that temporal ordering or causal mechanisms have been established.

### 5.1 Perceived academic stress and reactive aggression

This study found that, after controlling for sex, age, and grade, higher PAS was significantly and positively associated with higher RA, supporting H1. This finding is consistent with prior evidence that academic stress or educational stress is associated with higher levels of aggression, anger, and hostility among students (Bratu, 2024; Sengupta, 2023). It also aligns with research showing that adolescents with higher aggressiveness profiles may report greater stress exposure (Ruiz-Esteban et al., 2025), and with broader evidence linking stress to aggressive behavior through factors such as poor sleep quality and disrupted social bonds (Lai et al., 2025). In addition, perceived unfairness in school relationships has been found to prospectively predict subsequent reactive aggression, further suggesting that school-induced frustrating experiences are important for understanding adolescents’ aggressive responses (Gini et al., 2024). The present finding extends this literature by locating the stress–aggression association in the specific context of Chinese senior high school students, for whom coursework burden, grade comparison, adult evaluation, and competition for further education are highly salient. From the perspective of the frustration–aggression hypothesis, academic stress may be particularly relevant to RA when students appraise academic demands as persistent goal obstruction, evaluative threat, or failure-related frustration. In this sense, PAS does not simply reflect a heavy learning workload; it may represent a high-arousal and highly evaluative stress experience associated with externalizing responses under provocative conditions. This interpretation is also consistent with broader stress–aggression research, which suggests that adolescents’ aggressive tendencies are generally higher when they are chronically exposed to negative life events or high-pressure contexts (Yang et al., 2023).

The boundary of this interpretation should also be made clear. The PAS–RA association may vary according to the source and appraisal of academic stress and the broader school relational context; stressors perceived as threatening, unfair, or uncontrollable may be more strongly associated with reactive aggressive responses. Accordingly, higher PAS may warrant attention as one potential correlate of emotional and behavioral risk rather than as a stand-alone screening indicator. Future longitudinal and experience-sampling studies should examine whether the PAS–RA association varies across stress sources, appraisals, and school contexts.

### 5.2 Independent mediating effect of anger rumination

This study found a significant statistical indirect effect involving AR in the association between PAS and RA, supporting H2. Because the direct association remained significant after AR was included, AR should be understood as one non-exclusive statistical pathway. Existing research provides converging evidence for this interpretation. Stressful or victimizing experiences in school may become internalized as anger rumination and be associated with subsequent aggression risk. For example, peer victimization has been reported to predict subsequent increases in anger rumination (Spyropoulou and Giovazolias, 2023), and victimization experiences may be further associated with later relational aggression through anger rumination (Li et al., 2021). These findings are consistent with the perseverative cognition hypothesis, which suggests that stressful experiences do not necessarily end psychologically when the external event has passed; rather, they may persist through repeated cognitive processing. In the present context, higher PAS may be associated with more frequent recall and reinterpretation of stress-related experiences, consistent with higher AR. The downstream part of this pathway is also consistent with prior aggression research. AR has been reported to statistically mediate the association between trait anger and reactive aggression (Quan et al., 2022), and it may help characterize the link between anger and reactive aggression in longitudinal and experimental contexts, with self-immersed anger rumination posing greater risk than self-distanced processing (Wen et al., 2024). In addition, AR remains significantly associated with reactive aggression after controlling for proactive aggression (White and Turner, 2014), and it can function as a common cognitive–emotional pathway through which different antecedent variables are associated with aggression (García-Sancho et al., 2016). More direct experimental evidence further indicates that rumination after provocation increases rather than alleviates aggression (Bushman, 2002). Together, these findings suggest that AR may be especially relevant to RA because it maintains hostile cues, negative affect, and arousal, which are central components of the General Aggression Model.

The AR-related indirect association may vary across stressors and processing styles; it may be more salient when academic stress is appraised as unfair, humiliating, or threatening and when anger is processed in a self-immersed rather than self-distanced manner. Because the direct PAS–RA association remained significant, AR represents one non-exclusive statistical pathway rather than a complete explanation. Anger-related perseverative processing may therefore warrant consideration in future school-based intervention research, although the present study did not test whether modifying such processing reduces reactive aggression. Future longitudinal, experience-sampling, and experimental studies should examine whether PAS–AR–RA associations vary across stress sources and forms of anger rumination.

### 5.3 Independent mediating effect of self-control

This study found a significant statistical indirect effect involving SC in the association between PAS and RA, supporting H3. Because the direct PAS–RA association remained significant after SC was included, the indirect association involving lower SC was partial and did not fully account for the PAS–RA association. This finding is consistent with research suggesting that academic stress may be associated with lower self-control and that lower self-control may be associated with higher reactive aggression risk. From the perspective of the strength model of self-control, sustained academic demands may be associated with poorer subsequent self-control performance, as suggested by research conducted in examination contexts (Baumeister et al., 2007; Oaten and Cheng, 2005). However, ego-depletion effects remain debated: meta-analytic reanalysis and a preregistered multilab replication have questioned their robustness, while process-based accounts emphasize shifts in motivation and attention rather than resource loss alone (Carter and McCullough, 2014; Hagger et al., 2016; Inzlicht and Schmeichel, 2012). A longitudinal study of Korean adolescents further found that academic stress and self-control were involved in pathways related to pathological gaming, a gaming-related problem behavior (Jeong et al., 2019). In a sample of Chinese senior high school students, academic stress was also linked to components of executive function in a longitudinal network framework (Ma et al., 2025). The link between SC and RA can also be interpreted through the Instigation–Impellance–Inhibition Theory (I^3^ Theory). In provocative, frustrating, or high-arousal situations, weaker inhibition may make it more difficult to suppress immediate retaliatory impulses, thereby making RA more likely. Consistent with this view, research on Chinese senior high school students has shown that higher self-control is significantly associated with lower reactive aggression (Li Q. et al., 2023). Recent research using reactive aggression as the outcome has also indicated that self-control is involved in pathways associated with lower reactive aggression (Qu, 2025). In addition, longitudinal research has shown that executive control deficits are more likely to predict reactive aggression than proactive aggression (Rohlf et al., 2018), and that executive dysfunction may further increase adolescents’ reactive aggression through impulsivity (Su et al., 2024). These findings from related constructs are consistent with the downstream pathway examined in the present study.

The SC pathway should also be interpreted within appropriate boundaries. Academic stress is heterogeneous, and different sources of stress may not be equally associated with lower self-control. Moreover, self-control, executive functioning, and inhibitory control are closely related but not identical constructs. Thus, the PAS → SC → RA pathway may represent one part of a broader self-regulatory pattern rather than a complete explanation. Overall, PAS was directly statistically associated with RA and indirectly associated with higher RA through lower SC. These findings suggest that self-regulatory skills may warrant consideration as potential targets in future school-based intervention studies; however, the present design did not test whether strengthening such skills reduces reactive aggression. Future research should use longitudinal designs, behavioral tasks, and experience sampling to examine whether changes in SC temporally link PAS and RA.

### 5.4 Serial mediation effect of anger rumination and self-control

A significant serial statistical indirect effect involving AR and SC was observed in the association between PAS and RA, supporting H4. The observed pattern was consistent with the theory-driven statistical ordering of PAS, AR, SC, and RA, although the cross-sectional data cannot establish temporal sequence. Persistent anger rumination is not merely a temporary recall of emotional experiences; theoretically, it may reflect sustained cognitive processing associated with negative arousal and lower self-regulatory functioning. Experimental research has shown that higher levels of rumination are associated with greater impairment in controlled resource allocation (Levens et al., 2009). A multisystem model also suggests that angry rumination can amplify aggression risk across cognitive, emotional, physiological, and control-related domains (Denson, 2013). At the same time, although angry rumination has been shown to increase subjective anger, its association with behavioral inhibitory control is not stable across tasks, suggesting that the AR–control relation may be context- and measurement-dependent (Lievaart et al., 2017). In the present context, the S-REF Model provides a theoretical lens for the observed association between sustained anger-related thinking and lower SC. The relevance of PAS to this pattern may lie in its persistent and highly evaluative nature. Previous research has shown that academic stress is associated with academic burnout through rumination-related mechanisms (Zuo et al., 2024), which is consistent with the perseverative cognition hypothesis, namely that stressful events do not automatically dissipate once external circumstances end, but may be prolonged and maintained within the individual through perseverative cognitive processing.

Within the proposed statistical ordering, lower SC was further associated with higher RA. Prior research has linked lower control capacity to greater difficulty inhibiting aggressive impulses, particularly under conditions of provocation, frustration, or high arousal (DeWall et al., 2007; Finkel et al., 2009). In adolescent samples, higher self-control is generally associated with lower levels of anger, hostility, and aggression (Orkibi and Ronen, 2019). From an integrated cognitive perspective, hostile rumination, effortful control, and reactive aggression themselves constitute a mutually embedded cognitive–regulatory chain (Wilkowski and Robinson, 2010). Thus, within the theoretical framework of the present study, PAS can be viewed as an instigating stress source, AR as a cognitive–emotional processing pattern that may amplify aggressive tendencies, and lower SC as indicating weaker inhibitory functioning. According to the Instigation–Impellance–Inhibition Theory (I^3^ Theory), when instigating factors are strong, impelling forces increase, and inhibitory functioning is weaker, individuals are more likely to display reactive aggression. This serial indirect effect should not be overinterpreted: although statistically significant, it accounted for only 4.32% of the total effect, indicating a small component rather than a principal explanation of the PAS–RA association. The significant direct effect after both mediators were included further indicates that other cognitive, emotional, or interpersonal processes may also be involved. The theoretical implication is that anger-related perseverative processing and lower self-control may be understood as interrelated components of a broader stress–aggression association rather than as a complete causal chain. Future longitudinal and experience-sampling research should test the temporal relation between AR and SC and whether changes in these variables precede changes in RA.

## 6 Practical implications

This study offers preliminary, theory-informed implications for school-based mental health promotion and behavioral risk prevention by identifying an association pattern linking perceived academic stress (PAS), anger rumination (AR), self-control (SC), and reactive aggression (RA) among Chinese senior high school students. These statistical associations suggest that PAS, AR, and SC may warrant attention in future school-based support research, but they do not establish the effectiveness of any specific intervention or tiered prevention pathway.

At the school level, future intervention research could examine whether manageable academic demands and supportive, improvement-oriented feedback improve students’ stress-related emotional and behavioral adjustment. Students reporting high PAS may warrant additional emotional and behavioral support, although the present study did not evaluate any specific screening, classroom-management, or disciplinary approach.

Future school- and family-based studies could evaluate broad approaches to stress management, anger regulation, self-regulatory support, and supportive communication. Given the cross-sectional design, modest explanatory power, and small serial indirect effect, these suggestions should be treated as hypotheses for future longitudinal and intervention research rather than evidence for the effectiveness of any specific program.

## 7 Limitations and future directions

Several limitations should be noted. Although this study examined the association patterns among perceived academic stress (PAS), anger rumination (AR), self-control (SC), and reactive aggression (RA), as well as the independent and serial statistical mediation pathways involved in these associations, the findings should be interpreted in light of the following limitations.

### 7.1 Limitations of measurement methods

First, PAS, AR, SC, and RA were all measured using self-report scales at the same time point, which may have introduced social desirability bias, self-perception bias, and common-source bias. Although Harman’s single-factor test was conducted as a preliminary and limited diagnostic, it cannot rule out common-method variance. Although item content was retained, the original 4-point AR format and 3-point frequency-based RA format were adapted to 5-point agreement scales before data collection. Reliability estimates supported internal consistency, and CFA supported the prespecified factor structures in this sample; however, neither established score equivalence with the original response formats. Therefore, scores should not be directly compared across formats. In addition, no fixed calendar-based recall period was specified for AR or RA, which may have allowed respondents to use different temporal frames of reference. Future research should incorporate multi-informant or behavioral measures, directly compare adapted and original response formats, and use clearly defined recall periods.

### 7.2 Limitations in study design and causal inference

Second, this study used a cross-sectional design. Accordingly, the independent and serial statistical indirect effects involving AR and SC in the PAS–RA association should be interpreted as associations consistent with the proposed theoretical order rather than as evidence of temporal ordering or causal mechanisms. The serial ordering was theory-driven, and not all alternative orders or models were systematically compared; reverse or bidirectional associations therefore remain possible. Future longitudinal and experience-sampling studies should test temporal ordering and bidirectionality, whereas experimental studies should examine potential causal processes.

### 7.3 Limited adjustment for confounding factors

Third, although this study controlled for sex, age, and grade in the main models, other potential confounding factors may not have been sufficiently measured or adjusted for, such as family socioeconomic status, parental academic expectations, school climate, perceived teacher fairness, peer victimization, anxiety, depression, and trait anger. These factors may be simultaneously associated with PAS, AR, SC, and RA and may therefore have affected the estimated associations. Future research should include more relevant contextual and psychological variables and use hierarchical regression, multilevel modeling, or other appropriate analytical strategies to improve the robustness of the findings.

### 7.4 Model robustness and hypothesis testing

Fourth, although this study used confirmatory factor analysis to test the correlated four-construct measurement model and used PROCESS to examine the independent and serial statistical mediation pathways, limitations remain in model robustness. Specifically, this study did not systematically compare competing models, such as one-factor, two-factor, or three-factor models, nor did it conduct sensitivity tests for alternative serial mediation orders. In addition, measurement invariance across sex, age, and grade groups was not examined before group comparisons; therefore, the group differences should be interpreted with caution. Future research should combine competing model comparisons, alternative path testing, and latent variable structural equation modeling to improve the rigor of hypothesis testing.

### 7.5 Sample and generalizability

Fifth, the sample was drawn only from students at three senior high schools in Hengyang, Hunan Province, China, and therefore has clear regional, educational-contextual, and developmental-stage characteristics. In addition, participants may have been nested within schools or classes, but school- and class-level identifiers were not available in the analytic dataset; therefore, the effect of clustering on standard error estimation and significance testing could not be assessed directly. As a result, the findings should not be directly generalized to junior high school students, university students, or adolescents from other regions or cultural backgrounds. Future research should replicate these findings across broader regions, school types, and age groups, retain school- and class-level identifiers, and use multilevel models or cluster-robust standard errors to account for data clustering.

### 7.6 Future research directions and applications

Future research should further distinguish different sources of PAS, such as parental expectations, teacher evaluation, peer competition, and self-expectations, and examine their differential associations with AR, SC, and RA. Longitudinal, experience-sampling, and experimental studies are needed to test the temporal ordering among these variables, particularly between AR and SC. Finally, future intervention studies could evaluate whether school- and family-based approaches addressing academic stress, anger-related processing, and self-regulatory support improve students’ stress-related emotional and behavioral outcomes.

## 8 Conclusion

Perceived academic stress was positively associated with reactive aggression among Chinese senior high school students. Statistical indirect effects involving anger rumination and self-control were observed both separately and in the hypothesized serial ordering. These findings suggest that anger-related perseverative processing and lower self-control may be involved in the academic stress–reactive aggression association; however, the model’s modest explanatory power and the small serial indirect component warrant cautious interpretation. These findings provide a preliminary integrative perspective on school stress, anger-related cognition, self-control, and behavioral risk, but do not establish specific intervention targets.

Because this study used cross-sectional self-report data from three schools in one city, the findings cannot establish temporal ordering or causal relationships and should be generalized cautiously beyond the sampled context. Future longitudinal, experimental, experience-sampling, and multi-informant studies across more diverse settings should test the temporal ordering of these associations and evaluate potential school- and family-based support approaches.

## Data Availability

The data supporting the findings of this study are available from the corresponding author upon reasonable request, subject to applicable ethical and confidentiality considerations.

## References

[ref1] Agnew R. (1992). Foundation for a general strain theory of crime and delinquency. Criminology 30, 47–88. doi: 10.1111/j.1745-9125.1992.tb01093.x

[ref2] Allen J. J. Anderson C. A. Bushman B. J. (2018). The general aggression model. Curr. Opin. Psychol. 19, 75–80. doi: 10.1016/j.copsyc.2017.03.034, 29279227

[ref3] Anestis M. D. Anestis J. C. Selby E. A. Joiner T. E. (2009). Anger rumination across forms of aggression. Pers. Individ. Differ. 46, 192–196. doi: 10.1016/j.paid.2008.09.026

[ref4] Baumeister R. F. Vohs K. D. Tice D. M. (2007). The strength model of self-control. Curr. Dir. Psychol. Sci. 16, 351–355. doi: 10.1111/j.1467-8721.2007.00534.x

[ref5] Berkowitz L. (1989). Frustration-aggression hypothesis: examination and reformulation. Psychol. Bull. 106, 59–73. doi: 10.1037/0033-2909.106.1.59, 2667009

[ref6] Bratu I. (2024). Academic stress and aggression. The moderating role of social support. Studia Doctoralia 14, 96–105. doi: 10.47040/sdpsych.v14i2.165

[ref7] Brosschot J. F. Gerin W. Thayer J. F. (2006). The perseverative cognition hypothesis: a review of worry, prolonged stress-related physiological activation, and health. J. Psychosom. Res. 60, 113–124. doi: 10.1016/j.jpsychores.2005.06.074, 16439263

[ref8] Brosschot J. F. Verkuil B. Thayer J. F. (2010). Conscious and unconscious perseverative cognition: is a large part of prolonged physiological activity due to unconscious stress? J. Psychosom. Res. 69, 407–416. doi: 10.1016/j.jpsychores.2010.02.002, 20846542

[ref9] Bushman B. J. (2002). Does venting anger feed or extinguish the flame? Catharsis, rumination, distraction, anger, and aggressive responding. Personal. Soc. Psychol. Bull. 28, 724–731. doi: 10.1177/0146167202289002

[ref10] Carter E. C. McCullough M. E. (2014). Publication bias and the limited strength model of self-control: has the evidence for ego depletion been overestimated? Front. Psychol. 5:823. doi: 10.3389/fpsyg.2014.00823, 25126083 PMC4115664

[ref11] Chen H. Guo H. Chen H. Cao X. Liu J. Chen X. . (2023). Influence of academic stress and school bullying on self-harm behaviors among Chinese middle school students: the mediation effect of depression and anxiety. Front. Public Health 10:1049051. doi: 10.3389/fpubh.2022.1049051, 36684901 PMC9853286

[ref12] Chyu E. P. Y. Chen J.-K. (2022). The correlates of academic stress in Hong Kong. Int. J. Environ. Res. Public Health 19:4009. doi: 10.3390/ijerph19074009, 35409692 PMC8997729

[ref13] de Ridder D. T. D. Lensvelt-Mulders G. Finkenauer C. Stok F. M. Baumeister R. F. (2012). Taking stock of self-control: a meta-analysis of how trait self-control relates to a wide range of behaviors. Personal. Soc. Psychol. Rev. 16, 76–99. doi: 10.1177/1088868311418749, 21878607

[ref14] Denson T. F. (2013). The multiple systems model of angry rumination. Personal. Soc. Psychol. Rev. 17, 103–123. doi: 10.1177/1088868312467086, 23175519

[ref15] Denson T. F. DeWall C. N. Finkel E. J. (2012). Self-control and aggression. Curr. Dir. Psychol. Sci. 21, 20–25. doi: 10.1177/0963721411429451

[ref16] DeWall C. N. Baumeister R. F. Stillman T. F. Gailliot M. T. (2007). Violence restrained: effects of self-regulation and its depletion on aggression. J. Exp. Soc. Psychol. 43, 62–76. doi: 10.1016/j.jesp.2005.12.005

[ref17] DeWall C. N. Finkel E. J. Denson T. F. (2011). Self-control inhibits aggression. Soc. Personal. Psychol. Compass 5, 458–472. doi: 10.1111/j.1751-9004.2011.00363.x

[ref18] Finkel E. J. (2014). The I^3^ model: metatheory, theory, and evidence. Adv. Exp. Soc. Psychol. 49, 1–104. doi: 10.1016/B978-0-12-800052-6.00001-9

[ref19] Finkel E. J. DeWall C. N. Slotter E. B. Oaten M. Foshee V. A. (2009). Self-regulatory failure and intimate partner violence perpetration. J. Pers. Soc. Psychol. 97, 483–499. doi: 10.1037/a0015433, 19686003

[ref20] Finkel E. J. Hall A. N. (2018). The I^3^ model: a metatheoretical framework for understanding aggression. Curr. Opin. Psychol. 19, 125–130. doi: 10.1016/j.copsyc.2017.03.013, 29279210

[ref21] Gao X. (2023). Academic stress and academic burnout in adolescents: a moderated mediating model. Front. Psychol. 14:1133706. doi: 10.3389/fpsyg.2023.1133706, 37342640 PMC10278958

[ref22] García-Sancho E. Salguero J. M. Fernández-Berrocal P. (2016). Angry rumination as a mediator of the relationship between ability emotional intelligence and various types of aggression. Pers. Individ. Dif. 89, 143–147. doi: 10.1016/j.paid.2015.10.007

[ref23] Gini G. Angelini F. Pozzoli T. (2024). Unfair teachers, unhappy students: longitudinal associations of perceived teacher relational unfairness with adolescent peer aggression and school satisfaction. Front. Psychol. 15:1321050. doi: 10.3389/fpsyg.2024.1321050, 38708022 PMC11066656

[ref24] Hagger M. S. Chatzisarantis N. L. D. Alberts H. Anggono C. O. Batailler C. Birt A. R. . (2016). A multilab preregistered replication of the ego-depletion effect. Perspect. Psychol. Sci. 11, 546–573. doi: 10.1177/1745691616652873, 27474142

[ref25] Hagger M. S. Wood C. Stiff C. Chatzisarantis N. L. D. (2010). Ego depletion and the strength model of self-control: a meta-analysis. Psychol. Bull. 136, 495–525. doi: 10.1037/a0019486, 20565167

[ref26] Harmon S. L. Stephens H. F. Repper K. K. Driscoll K. A. Kistner J. A. (2019). Children's rumination to sadness and anger: implications for the development of depression and aggression. J. Clin. Child Adolesc. Psychol. 48, 622–632. doi: 10.1080/15374416.2017.1359788, 28841342

[ref27] Herts K. L. McLaughlin K. A. Hatzenbuehler M. L. (2012). Emotion dysregulation as a mechanism linking stress exposure to adolescent aggressive behavior. J. Abnorm. Child Psychol. 40, 1111–1122. doi: 10.1007/s10802-012-9629-4, 22466516 PMC3448707

[ref28] Hua Y. Huang C. Guo Y. Du X. Guo L. Wang W. . (2023). Association between academic pressure, *NR3C1* gene methylation, and anxiety symptoms among Chinese adolescents: a nested case-control study. BMC Psychiatry 23:376. doi: 10.1186/s12888-023-04816-7, 37254074 PMC10227793

[ref29] Inzlicht M. Schmeichel B. J. (2012). What is ego depletion? Toward a mechanistic revision of the resource model of self-control. Perspect. Psychol. Sci. 7, 450–463. doi: 10.1177/1745691612454134 26168503

[ref30] Jeong E. J. Ferguson C. J. Lee S. J. (2019). Pathological gaming in young adolescents: a longitudinal study focused on academic stress and self-control in South Korea. J. Youth Adolesc. 48, 2333–2342. doi: 10.1007/s10964-019-01065-4, 31327099

[ref31] Jiang M.-M. Gao K. Wu Z.-Y. Guo P.-P. (2022). The influence of academic pressure on adolescents' problem behavior: chain mediating effects of self-control, parent–child conflict, and subjective well-being. Front. Psychol. 13:954330. doi: 10.3389/fpsyg.2022.954330, 36211862 PMC9534181

[ref32] Kim J. A. Choi J. I. Lee S. J. Jeong E. J. (2025). The influence of negative emotions on adolescents' pathological gaming: the role of self-control in the associations with aggression, academic stress, and pathological gaming. Front. Psychol. 16:1539162. doi: 10.3389/fpsyg.2025.1539162, 40672844 PMC12263935

[ref33] Lai S. Li S. D. Huang Y. Chen L. (2025). The role of poor sleep quality and disrupted social bonds in stress-fueled aggressive behavior: a mixed-methods analysis. Aggress. Behav. 51:e70048. doi: 10.1002/ab.70048, 40985259

[ref34] Levens S. M. Muhtadie L. Gotlib I. H. (2009). Rumination and impaired resource allocation in depression. J. Abnorm. Psychol. 118, 757–766. doi: 10.1037/a0017206, 19899845 PMC2836938

[ref35] Li Q. Gao M. Deng Y. Zhou Z. Wang J. (2023). Parental attachment and proactive-reactive aggression in adolescence: the mediating role of self-control and perspective taking. Psychol. Res. Behav. Manag. 16, 3437–3446. doi: 10.2147/PRBM.S423270, 37664135 PMC10473417

[ref36] Li C. Zhang X. Cheng X. (2023). Associations among academic stress, anxiety, extracurricular participation, and aggression: an examination of the general strain theory in a sample of Chinese adolescents. Curr. Psychol. 42, 21351–21362. doi: 10.1007/s12144-022-03204-w

[ref37] Li C. Zhao Q. Dai W. Zhang Y. (2021). Victims become covert aggressors: gender differences in the mediating effects of rumination on anger and sadness. J. Psychol. 155, 441–456. doi: 10.1080/00223980.2021.1901254, 33830888

[ref38] Lievaart M. Huijding J. van der Veen F. M. Hovens J. E. Franken I. H. A. (2017). The impact of angry rumination on anger-primed cognitive control. J. Behav. Ther. Exp. Psychiatry 54, 135–142. doi: 10.1016/j.jbtep.2016.07.016, 27494341

[ref39] Luo S. Ban Y. Qiu T. Liu C. (2023). Effects of stress on school bullying behavior among secondary school students: moderating effects of gender and grade level. Front. Psychol. 14:1074476. doi: 10.3389/fpsyg.2023.1074476, 37008878 PMC10050707

[ref40] Ma C. Wang Y. Fu J. Zhao X. (2025). Longitudinal relationships between academic stress and executive function in Chinese high school students: a cross-lagged panel network analysis. Contemp. Educ. Psychol. 83:102424. doi: 10.1016/j.cedpsych.2025.102424

[ref41] Ministry of Education of the People’s Republic of China Supreme People’s Procuratorate of the People’s Republic of China Publicity Department of the CPC Central Committee Office of the Central Cyberspace Affairs Commission Ministry of Science and Technology of the People’s Republic of China Ministry of Public Security of the People’s Republic of China . (2023). Notice on the Issuance of the Special Action Plan for Comprehensively Strengthening and Improving Student Mental Health in the New Era (2023–2025) [in Chinese]. Available online at: https://www.moe.gov.cn/srcsite/A17/moe_943/moe_946/202305/t20230511_1059219.html (Accessed March 12, 2026).

[ref42] Moffitt T. E. Arseneault L. Belsky D. Dickson N. Hancox R. J. Harrington H. . (2011). A gradient of childhood self-control predicts health, wealth, and public safety. Proc. Natl. Acad. Sci. USA 108, 2693–2698. doi: 10.1073/pnas.1010076108, 21262822 PMC3041102

[ref43] Navas-Casado M. L. García-Sancho E. Salguero J. M. (2023). Associations between maladaptive and adaptive emotion regulation strategies and aggressive behavior: a systematic review. Aggress. Violent Behav. 71:101845. doi: 10.1016/j.avb.2023.101845

[ref44] Oaten M. Cheng K. (2005). Academic examination stress impairs self-control. J. Soc. Clin. Psychol. 24, 254–279. doi: 10.1521/jscp.24.2.254.62276

[ref45] Orkibi H. Ronen T. (2019). A dual-pathway model linking self-control skills to aggression in adolescents: happiness and time perspective as mediators. J. Happiness Stud. 20, 729–742. doi: 10.1007/s10902-018-9967-1

[ref46] Ottaviani C. Thayer J. F. Verkuil B. Lonigro A. Medea B. Couyoumdjian A. . (2016). Physiological concomitants of perseverative cognition: a systematic review and meta-analysis. Psychol. Bull. 142, 231–259. doi: 10.1037/bul0000036, 26689087

[ref47] Peled M. Moretti M. M. (2010). Ruminating on rumination: are rumination on anger and sadness differentially related to aggression and depressed mood. J. Psychopathol. Behav. Assess. 32, 108–117. doi: 10.1007/s10862-009-9136-2

[ref48] Podsakoff P. M. MacKenzie S. B. Lee J.-Y. Podsakoff N. P. (2003). Common method biases in behavioral research: a critical review of the literature and recommended remedies. J. Appl. Psychol. 88, 879–903. doi: 10.1037/0021-9010.88.5.879, 14516251

[ref49] Qu Y. (2025). The relationship between dispositional awe and reactive aggression: the serial mediation role of trait anger and self-control. Psychol. Rep. 128, 1024–1041. doi: 10.1177/00332941231168558, 37012025

[ref50] Quach A. S. Epstein N. B. Riley P. J. Falconier M. K. Fang X. (2015). Effects of parental warmth and academic pressure on anxiety and depression symptoms in Chinese adolescents. J. Child Fam. Stud. 24, 106–116. doi: 10.1007/s10826-013-9818-y

[ref51] Quan F. Wang L. Gong X. Lei X. Liang B. Zhang S. (2022). Hostile attribution bias and anger rumination sequentially mediate the association between trait anger and reactive aggression. Front. Psychol. 12:778695. doi: 10.3389/fpsyg.2021.778695, 35095661 PMC8790055

[ref52] Raine A. Dodge K. A. Loeber R. Gatzke-Kopp L. M. Lynam D. R. Reynolds C. . (2006). The reactive–proactive aggression questionnaire: differential correlates of reactive and proactive aggression in adolescent boys. Aggress. Behav. 32, 159–171. doi: 10.1002/ab.20115, 20798781 PMC2927832

[ref53] Rohlf H. L. Holl A. K. Kirsch F. Krahé B. Elsner B. (2018). Longitudinal links between executive function, anger, and aggression in middle childhood. Front. Behav. Neurosci. 12:27. doi: 10.3389/fnbeh.2018.00027, 29535615 PMC5835083

[ref54] Ruiz-Esteban C. Méndez I. Martínez-Ramón J. P. Antón-Ros N. Lagos San Martín N. G. (2025). Profiles of aggressiveness and stress in Spanish adolescents. Eur. J. Investig. Health Psychol. Educ. 15:112. doi: 10.3390/ejihpe15060112, 40558854 PMC12192317

[ref55] Sengupta R. (2023). Effect of academic stress, loneliness and life satisfaction on aggression levels of students: a COVID-19 context. Int. J. Adv. Res. 11, 472–480. doi: 10.21474/IJAR01/17412

[ref56] Smith S. D. Stephens H. F. Repper K. Kistner J. A. (2016). The relationship between anger rumination and aggression in typically developing children and high-risk adolescents. J. Psychopathol. Behav. Assess. 38, 515–527. doi: 10.1007/s10862-016-9542-1

[ref57] Spyropoulou E. Giovazolias T. (2023). Exploring the longitudinal relationship between anger rumination and peer victimization when controlling for sadness rumination. Curr. Psychol. 42, 28263–28275. doi: 10.1007/s12144-022-03906-1

[ref58] Steare T. Gutiérrez Muñoz C. Sullivan A. Lewis G. (2023). The association between academic pressure and adolescent mental health problems: a systematic review. J. Affect. Disord. 339, 302–317. doi: 10.1016/j.jad.2023.07.028, 37437728

[ref59] Su X. Li W. Hu C. Liu H. Lian R. (2024). The longitudinal relationship between executive dysfunction and reactive and proactive aggression in adolescents: impulsivity as a mediator and sex differences. Front. Psych. 15:1484340. doi: 10.3389/fpsyt.2024.1484340, 39758445 PMC11695426

[ref60] Sukhodolsky D. G. Golub A. Cromwell E. N. (2001). Development and validation of the anger rumination scale. Pers. Individ. Dif. 31, 689–700. doi: 10.1016/S0191-8869(00)00171-9

[ref61] Sun J. Dunne M. P. Hou X.-Y. Xu A.-Q. (2011). Educational stress scale for adolescents: development, validity, and reliability with Chinese students. J. Psychoeduc. Assess. 29, 534–546. doi: 10.1177/0734282910394976

[ref62] Tangney J. P. Baumeister R. F. Boone A. L. (2004). High self-control predicts good adjustment, less pathology, better grades, and interpersonal success. J. Pers. 72, 271–324. doi: 10.1111/j.0022-3506.2004.00263.x, 15016066

[ref63] Tuvblad C. Dhamija D. Berntsen L. Raine A. Liu J. (2016). Cross-cultural validation of the reactive-proactive aggression questionnaire (RPQ) using four large samples from the US, Hong Kong, and China. J. Psychopathol. Behav. Assess. 38, 48–55. doi: 10.1007/s10862-015-9501-2, 27330246 PMC4908966

[ref64] Unger A. Bi C. Xiao Y.-Y. Ybarra O. (2016). The revising of the Tangney self-control scale for Chinese students. PsyCh J. 5, 101–116. doi: 10.1002/pchj.128, 27144924

[ref65] Wang J. Wang Z. Yang Y. Wang T. Lin H. Zhang W. . (2025). Academic burden and emotional problems among adolescents: a longitudinal mediation analysis. J. Adolesc. 97, 989–1001. doi: 10.1002/jad.12471, 39835663 PMC12128909

[ref66] Wells A. Matthews G. (1996). Modelling cognition in emotional disorder: the S-REF model. Behav. Res. Ther. 34, 881–888. doi: 10.1016/S0005-7967(96)00050-2, 8990539

[ref67] Wen J. Wang G. Miao M. (2024). The link between anger and reactive aggression: insights into anger rumination. Aggress. Behav. 50:e22157. doi: 10.1002/ab.22157, 38770707

[ref68] White B. A. Turner K. A. (2014). Anger rumination and effortful control: mediation effects on reactive but not proactive aggression. Pers. Individ. Dif. 56, 186–189. doi: 10.1016/j.paid.2013.08.012

[ref69] Wilkowski B. M. Robinson M. D. (2010). The anatomy of anger: an integrative cognitive model of trait anger and reactive aggression. J. Pers. 78, 9–38. doi: 10.1111/j.1467-6494.2009.00607.x, 20433611

[ref70] Williams D. P. Feeling N. R. Hill L. K. Spangler D. P. Koenig J. Thayer J. F. (2017). Resting heart rate variability, facets of rumination and trait anxiety: implications for the perseverative cognition hypothesis. Front. Hum. Neurosci. 11:520. doi: 10.3389/fnhum.2017.00520, 29163100 PMC5671536

[ref71] Wuthrich V. M. Jagiello T. Azzi V. (2020). Academic stress in the final years of school: a systematic literature review. Child Psychiatry Hum. Dev. 51, 986–1015. doi: 10.1007/s10578-020-00981-y, 32180075

[ref72] Xiong M. Wang Q. Xiong Y. Jiang W. Ren P. (2024). The effect of adolescents' perception of parental academic pressure on psychological adaptation: the protective role of ego-resilience [in Chinese]. Psychol. Dev. Educ. 40, 542–550. doi: 10.16187/j.cnki.issn1001-4918.2024.04.09

[ref73] Yang S. L. Tan C. X. Li J. Zhang J. Chen Y. P. Li Y. F. . (2023). Negative life events and aggression among Chinese rural left-behind adolescents: do self-esteem and resilience mediate the relationship? BMC Psychiatry 23:167. doi: 10.1186/s12888-023-04587-1, 36922776 PMC10015683

[ref74] Ye B. Wu D. Im H. Liu M. Wang X. Yang Q. (2020). Stressors of COVID-19 and stress consequences: the mediating role of rumination and the moderating role of psychological support. Child Youth Serv. Rev. 118:105466. doi: 10.1016/j.childyouth.2020.105466, 32994656 PMC7515821

[ref75] Zhu M. Tu T. (2025). Associations between parental psychological control and negative emotions in early adolescents: a moderated mediation model. Front. Psychol. 16:1618832. doi: 10.3389/fpsyg.2025.1618832, 41040094 PMC12484177

[ref76] Zuo X. Zhao L. Li Y. He W. Yu C. Wang Z. (2024). Psychological mechanisms of English academic stress and academic burnout: the mediating role of rumination and moderating effect of neuroticism. Front. Psychol. 15:1309210. doi: 10.3389/fpsyg.2024.1309210, 38328384 PMC10847527

